# New hybrid EC-PROMETHEE method with multiple iterations of random weight ranges: Step-by-step application in Python

**DOI:** 10.1016/j.mex.2024.102890

**Published:** 2024-08-05

**Authors:** Marcio Pereira Basilio, Valdecy Pereira, Fatih Yiğit

**Affiliations:** a Controladoria-Geral do Estado do Rio de Janeiro (CGE), Avenida Erasmo Braga, 118, Centro, Rio de Janeiro 20020-000, Brazil; b Department of Production Engineering, Fluminense Federal University (UFF), Niteroi 24210-240, Brazil; c Department of Industrial Engineering, Altinbas University, Istanbul 34218, Turkey

**Keywords:** Entropy, Critic, Promethee, Decision maker, Mcda, Operations research, EC-PROMETHEE

## Abstract

The decision-making process consists of finding the best solution to an analyzed problem. This search is carried out in the face of countless interactions when analyzing an alternative criterion by criterion, under which weights are assigned that distinguish the degree of importance they have for the decision-makers. The definition of weight for each criterion gives rise to three lines of thought on the subject. There are objective, subjective, and hybrid methods. This discussion concerns the degree to which experts define the criteria weights. Based on this discussion, we developed a hybrid method to integrate the Entropy and CRITIC methods with the PROMETHEE method, called EC-PROMETHEE. The innovation of this method is that the combination of the Entropy and CRITIC methods does not result in a single set of weights. In reality, the weights generated by each method are used to define each criterion's upper and lower limits. The range of weights generated for each criterion is emulated "n" times and builds a set of weights that are applied to the ranking definition process. The model generates "n" rankings, defining a single ranking. In this article, we demonstrate a step-by-step application of a tool developed in Python called EC-PROMETHEE and use it as an example of the problem of choosing rotary-wing airplanes for application in the military police service.

➢ *The method reduces discretion in determining the weights of the criteria;*
➢ *The innovation lies in the use of a range of weights for criteria;*
➢ *Consistency in defining the final ranking.*

## Graphical abstract

**Figure:**
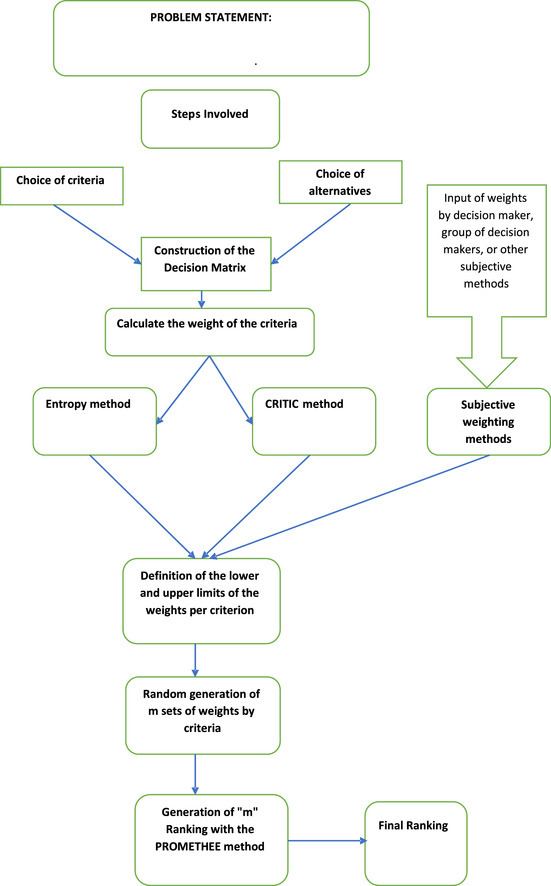

Specifications table

**Table:** 

Subject area:	Engineering
More specific subject area:	*Decision Science*
Name of your method:	EC-PROMETHEE
Name and reference of original method:	Basilio, M.P.; Pereira, V.; Yigit, F. New Hybrid EC-Promethee Method with Multiple Iterations of Random Weight Ranges: Applied to the Choice of Policing Strategies. Mathematics 2023, 11, 4432. 10.3390/math11214432]
Resource availability:	ec-promethee PyPI]

## Background

Decision-making is an inherently human activity; we do it thousands of times a day, both consciously and unconsciously. We can distinguish between simple decisions, such as choosing what color suit to wear, whether to take an umbrella or not, or whether to travel to work by car or metro. At the other end of the spectrum are complex decisions, such as defining public policies, investments, company mergers, etc. I want to clarify that decisions are made daily and in various fields of knowledge. In this sense, decision science has been widely studied over the last 60 years, mainly in the field of operational research. Basilio et al. [1] revealed in their research the evolution of multi-criteria methods, which have been developed over the decades to support decision-making. The use of multi-criteria methods by decision-makers is justified by the limitation of human rationality in quickly evaluating and distinguishing the best alternative solution to a problem from among N alternatives submitted to a set of M criteria. In this judgment process, we also have the value of the decision maker's perception of each criterion, which we define as the weight of the criteria. This is another discussion in which numerous methods for defining criteria weights have been suggested. Ayan et al. [2] presented a set of these methods in their work, among which we can state that the AHP [1,3] is the method most researchers use when integrating methods for measuring weights with methods for ordering alternatives. This is followed by DEMATEL [4], SWARA [[5], [6], [7]], ANP [4], ENTROPY [8], CRITIC [9], BWM [10], CILOS [11], IDOCRIW [11], FUCOM [12,13], LBWA [14], SAPEVO-M [15], MEREC [16,17], LOPCOW [18], and RAFSI [19].

The space created between experts who advocate using objective methods for obtaining criteria weights removes human intervention from the process, thus trying to eliminate the subjectivity behind the definition of criteria weights. Some advocate only human intervention in this process, as it brings the experience of specialists and intrinsic knowledge of the problems to be solved into the process. However, it can also bring negative subjectivities such as private, corporate, and political interests favoring a particular solution. As a result, a third branch of this discussion emerged: hybrid methods, which seek a balance by combining the weights of criteria obtained by objective and subjective methods. Based on these discussions, Basilio et al. [20] developed the EC-PROMETHEE method, integrating the objective ENTROPY and CRITIC methods with the multi-criteria PROMETHEE method. This new method combines objective and subjective methods for obtaining the weight of the criteria and the possibility of inserting a third external method, which can be objective or subjective. The innovation contained in this method lies in the creation of a weight range for each criterion, preserving the characteristics of each technique. This technique differs from other hybrid methods in that it is not an algebraic combination of the different methods used. In this sense, each weight range comprises lower and upper limits, which can be combined to generate random numbers, producing "t" sets of weights per criterion, making obtaining "t" final classifications possible. The alternatives receive a value corresponding to their position in each ranking generated. At the end of the process, they will be ranked in descending order, thus obtaining the final definitive ranking. In this way, managers can analyse the behaviour of each alternative throughout the process, and the final ranking will be more consistent due to the incorporation of the variations caused by the influence of the weight of the criteria on the alternatives.

This article is structured in three distinct sections. The first is what we call Background, where we introduce the main concepts used throughout the text. Next, we have the Method details. In this section, the EC-PROMETHEE method has been described in all its phases. We come to Method validation. In this section we will demonstrate the step-by-step of the EC_PROMETHEE method through the tool developed in Python. Finally, We have a short section called Final Considerations. Where we summarize the main points of the article.

## Method details

**Fig. 1:**
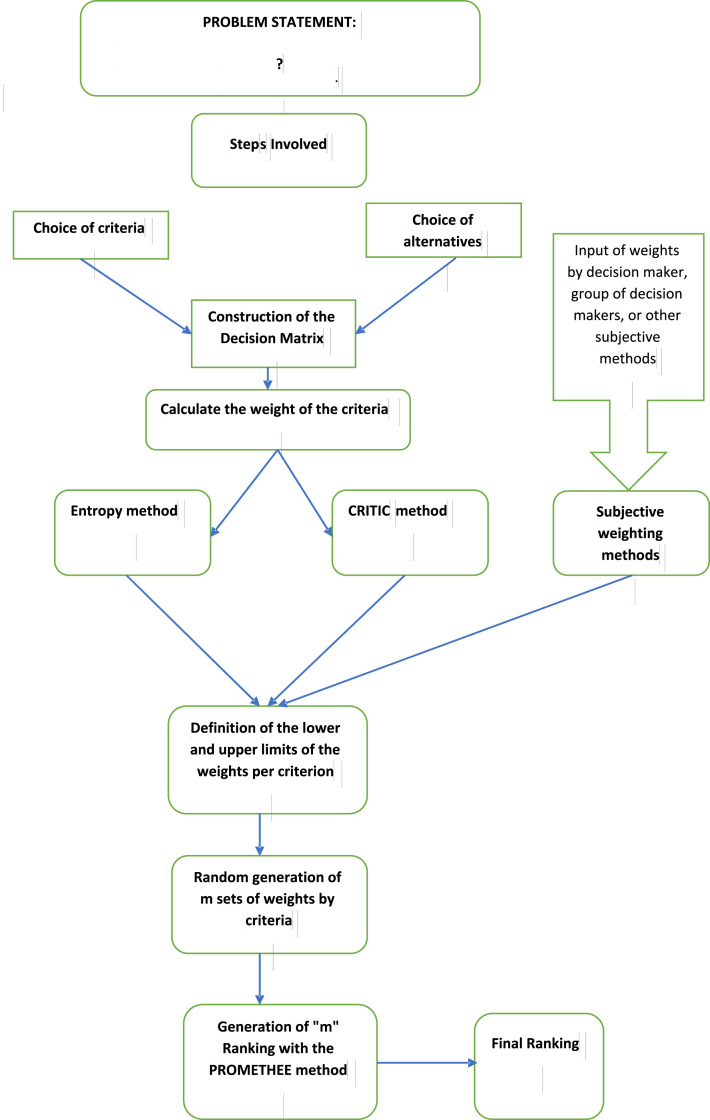
Methodological scheme. *Note*: based on de Basilio et al. [20].

This section presents the concepts and formulations in formulating the hybrid EC-PROMETHEE method. Fig. 1 illustrates the description of the proposed method by subdividing it into eight steps. Fig. 1 can be applied to any type of problem in which the decision maker needs to rank alternatives, i.e., a generic problem-solving scheme. For example, the seminal paper by Basilio et al. [20] addressed the problem of ranking policing strategies.

Step 1 – Identification of criteria

At this stage, the decision-makers and/or analysts of the problem under study identify the criteria that will be included in the model to solve the problem.

Step 2 – Identification of alternative

At this stage, the decision-makers and/or analysts of the problem under study identify the alternatives that will be included in the model to solve the problem.

Step 3 – Construction of the decision matrix

At this stage, the decision-makers and/or analysts of the problem under study collect the information corresponding to the alternatives criterion by criterion, which will be inserted into the model to solve the problem.

Step 4 Description of how the criteria weights are obtained

Step 4.1 The ENTROPY method

The criteria weights are based on a predefined decision matrix (DM) comprising information for the set of candidate materials when the Entropy method is used. Entropy in information theory is a model for the uncertainty volume served by a discrete probability distribution [22]. Salwa et al. [23] used the entropy method to calculate criterion weight to select optimal starch as the matrix in green composites for single-use food packaging applications [23]. The Entropy of the normalized decision matrix (NDM) criterion is given in Eq. (1):

(1) $$E_{j}=\;-\;\frac{\left[\sum_{i=1}^{m}P_{ij}\text{ln}\left(P_{ij}\right)\right]}{\text{ln}\left(m\right)};j=1,2,\ldots ,n\;and\;i=1,2,\ldots ,m$$

where $P_{ij}$ is NDM, which is given by Eq. (2):

(2) $$P_{ij}=\frac{x_{ij}}{\sum_{i=1}^{m}x_{ij}};j=1,2,\ldots ,n\;and\;i=1,2,\ldots ,m$$

where $x_{ij}$ corresponds to the criteria value for each alternative in DM. The criteria weight,

$W_{j}^{E}\;$can be calculated using Eq. (3):

(3) $$W_{j}^{E}=\frac{1-E_{j}}{\sum_{j=1}^{n}1-E_{j}};j=1,2,\ldots ,n$$

where $\left(1-E_{j}\right)$ denotes the degree of diversity of the information in the jth criterion outcome.

Step 4.2 The CRITIC method

In this section, a brief description of the CRITIC method is presented. The CRITIC method proposed by [21] aims to determine the criteria weights. In this method, the qualitative attributes are replaced with some quantities, and the independence of the attributes is not obligatory. The main steps of this technique can be described as follows:

Step 4.2.1. A decision matrix, Z, with m rows as the number of alternatives and n column as the number of criteria, is defined by Eq. (4):

(4) $$Z={\left(r_{ij}\right)}_{mxn};\;i=1,\ldots ,m;j=1,\ldots ,n$$

where $r_{ij}$ is the correlation of the ith alternative and the jth criterion.

Step 4.2.2. Each criterion can be considered beneficial or non-beneficial [[24], [25], [26]]. A criterion takes value in some bounded range. For a beneficial one,$j\;\in F^{+}$, the criterion is normalized by dividing its distance from the minimum value by the length of the range. In contrast, a non-beneficial one, $\in F^{-}$, is normalized by dividing its distance from the maximum value by the length of the range. The elements of the decision matrix are normalized as given in Eqs. (5),(6) for the positive or beneficial criteria and the negative or non-beneficial ones.

(5) $$x_{ij}^{+}=\frac{r_{ij}-r_{j}^{-}}{r_{j}^{+}-r_{j}^{-}};\;i=1,\ldots ,m;j=1,\ldots ,n\;if\;j\;\in \;F^{+}$$

(6) $$x_{ij}^{-}=\frac{r_{j}^{+}-r_{ij}}{r_{j}^{+}-r_{j}^{-}};\;i=1,\ldots ,m;j=1,\ldots ,n\;if\;j\;\in \;F^{-}$$

where $r_{j}^{+}=\text{max}\left(r_{1j},r_{2j},\ldots ,r_{mj}\right)$ and $r_{j}^{-}=\text{min}\left(r_{1j},r_{2j},\ldots ,r_{mj}\right)$, and $x_{ij}$ which is either $x_{j}^{+}$ or $x_{j}^{-}$represents the normalized value of the ij element of the decision matrix.

Step 4.2.3. The Pearson correlation coefficient between two criteria, j and k, is computed as Eq. (7)

(7) $$\rho_{jk}=\frac{\sum_{i=1}^{m}\left(x_{ij}-\underset{‾}{x_{j}}\right)\left(x_{ik}-\underset{‾}{x_{k}}\right)}{\sqrt{\sum_{i=1}^{m}{\left(x_{ij}-\underset{‾}{x_{j}}\right)}^{2}\sum_{i=1}^{m}{\left(x_{ik}-\underset{‾}{x_{k}}\right)}^{2}}}$$

where $\underset{‾}{x_{j}}$ and $\underset{‾}{x_{k}}$ represent the mean of *jth* and *kth* criteria Eq. (8):

(8) $$\underset{‾}{x_{k}}=\frac{1}{n}\sum_{i=1}^{m}x_{ik};\;k=1,\ldots ,n.$$

The Pearson correlation coefficient captures linear correlations.

Step 4.2.4. The standard deviation of each criterion is estimated by Eq. (9):

(9) $$\sigma_{j}=\sqrt{\frac{1}{n-1}\sum_{i=1}^{m}{\left(x_{ij}-\underset{‾}{x_{j}}\right)}^{2};}\;j=1,\ldots ,n$$

Step 4.2.5. The index of the jth criteria, Ej, is evaluated by Eq. (10)

(10) $$E_{j}=\sigma_{j}\sum_{k=1}^{n}\left(1-\rho_{jk}\right);\;j=1,\ldots ,n.$$

Step 4.2.6. The weights of the criteria are determined by Eq. (11)

(11) $$W_{j}^{C}=\frac{E_{j}}{\sum_{j=1}^{n}E_{j}};\;j=1,\ldots ,n.$$

Finally, the ranking of the weights of the criteria is obtained. The ranking identifies the importance given to each criterion.

Step 5 – Definition of the lower and upper limits of the weights per criterion

After generating the weights of each criterion using the Entropy and CRITIC methods, which constitute the objective methods, the model opens the door to input weights from subjective methods, which can be obtained by a single decision maker or a group of decision-makers, with or without the use of subjective methods [27] such as AHP; SAPEVO-M; FUCOM; MEREC among others.

In this step, we define the lower-limit vector. $Ll_{j}$ where criterion j will store the smallest weight value obtained from the set of values formed by $\left\{W_{j}^{E},\;W_{j}^{C},W_{j}^{DM}\right\}$, as shown in Eq. (12)

(12) $$Ll_{j}=Min\left\{W_{j}^{E},\;W_{j}^{C},W_{j}^{DM}\right\}$$

Next, we will define the upper limit vector. $Ul_{j}$, which for each criterion j will store the highest weight value obtained from the set of values formed by $\left\{W_{j}^{E},\;W_{j}^{C},W_{j}^{DM}\right\}$, as shown in Eq. (13)

(13) $$Ul_{j}=Max\left\{W_{j}^{E},\;W_{j}^{C},W_{j}^{DM}\right\}$$

It should be made clear to the reader that the variable $W_{j}^{DM}$ corresponds to the insertion of the weight from outside the model, as shown in Fig. 1. This insertion can come from subjective weights from the decision-makers, or group of decision-makers, or just the input of other objective weight generation methods, such as the AHP. In the event that no external data is entered, the model will assume that $W_{j}^{DM}=1\;\forall \;j=1,\ldots ,n.$

Step 6 – Random generation of "t" sets of weights by criteria

In this phase, the Randomised Weight Matrix RWm of dimension t x n will be generated. This stage is one of the model's innovations. In the cases found in the literature, when more than one method is used to obtain the weights of the criteria, mathematical operations are usually carried out to obtain a single set of weights for the criteria. In this model, RWm will allow you to obtain t sets of weights, which when applied to the PROMETHEE method will result in t final rankings. In reality, this process corresponds to a sensitivity analysis included in the model. Where t is the total number of rows, corresponding to the total number of iterations inserted in the model by the decision maker. Where n is the total number of columns of the matrix. The RWm matrix is obtained by generating different random numbers limited for each criterion by the limits. $Ll_{j}$ and $Ul_{j}$, as shown in Eq. (14):

(14) $$RWm_{ij}=\left(\left(Ul_{j}\;-\;Ll_{j}\right)*Rnd\right)+Ll_{j})$$

Next, the matrix $RWm_{ij}$ is normalized by Eq. (15):

(15) $$RWm_{ij}^{n}=\;\frac{x_{ij}}{\sum_{j=1}^{n}x_{ij}}$$

Step 7 – Generation of "t" Ranking with the PROMETHEE method

The method is implemented in five steps. In the first step, there is a function showing the decision-maker's preference concerning share "a" compared with share "b". The second step compares the suggested alternatives to the pairs for the preference function. The PROMETHEE proposes the six following types (shapes) of preference functions, as shown in Table 1:

**Table 1:** Types of preference function.

Type	Generalized criterion	Condition	Quantification of preference	Parameter to fix
Type I – Usual preference function		g(a)−g(b)>0 g(a)−g(b)≤0	$P_{j}\left(a,b\right)=1$ $P_{j}\left(a,b\right)=0$	–
Type II – U-shape preference function		g(a)−g(b)>q g(a)−g(b)≤q	$P_{j}\left(a,b\right)=1$ $P_{j}\left(a,b\right)=0$	q
Type III – V-shape preference function		g(a)−g(b)>p g(a)−g(b)≤p g(a)−g(b)≤0	$P_{j}\left(a,b\right)=1$ $P_{j}\left(a,b\right)=\frac{\left[g(a)-g(b)\right]}{p}$ $P_{j}\left(a,b\right)=0$	p
Type IV – Level preference function		|g(a)−g(b)|>p q<|g(a)−g(b)|≤p |g(a)−g(b)|≤q	$P_{j}\left(a,b\right)=1$ $P_{j}\left(a,b\right)=\frac{1}{2}$ $P_{j}\left(a,b\right)=0$	p,q
Type V – Linear preference function		|g(a)−g(b)|>p q<|g(a)−g(b)|≤p |g(a)−g(b)|≤q	$P_{j}\left(a,b\right)=1$ $P_{j}\left(a,b\right)=\frac{\left[|g(a)-g(b)|-q\right]}{\left(p-q\right)}$ $P_{j}\left(a,b\right)=0$	p,q
Type VI – Gaussian preference function		g(a)−g(b)>0 g(a)−g(b)≤0	$P_{j}\left(a,b\right)=1-e^{\left\{\frac{-{\left(g(a)-g(b)\right)}^{2}}{2s^{2}}\right\}}$ $P_{j}\left(a,b\right)=0$	s

*Note: based on de Basilio et al. [**20**].*

As a third step, the results of this comparison are presented in an evaluation matrix as the estimated values of each criterion for each alternative. The classification is performed in two final steps: a partial ranking in the fourth stage and then a total ranking of alternatives in the fifth step, as follows:

Step 7.1. Determination of deviations based on pair-wise comparations

(16) $$d_{j}\left(a,\;b\right)=g_{j}\left(a\right)-g_{j}\left(b\right)$$

Where $d_{j}\left(a,\;b\right)$ denotes the difference between the evaluations of a and b on each criterion.

Step 7.2. Application of the preference function

(17) $$P_{j}\left(a,b\right)=F_{j}\left[d_{j}\left(a,\;b\right)\right]\;j=1,\ldots ,\;k$$

Where $P_{j}\left(a,b\right)$ denotes the preference of alternative a with regard to alternative b on each criterion as a function of $d_{j}\left(a,\;b\right)$.

Step 7.3. Calculation of an overall or global preference index

(18) $$\forall \;a,\;b\;\varepsilon \;A,\;\pi \left(a,\;b\right)=\sum_{j=1}^{k}P_{j}\left(a,\;b\right)w_{j}$$

Where π(a,b) of *a* over b is defined as the weighted sum $P_{j}\left(a,\;b\right)$ of for each criterion and $w_{j}$ is the weight associated with the jth criterion.

Step 7.4. Calculation of outranking flows/ The PROMETHEE II partial ranking

(19) $$\varphi^{+}\left(a\right)=\sum_{x\;\in A}\pi \left(a,\;b\right)$$

And

(20) $$\varphi^{-}\left(a\right)=\sum_{x\;\in A}\pi \left(b,\;a\right)$$

Where $\varphi^{+}\left(a\right)$ and $\varphi^{-}\left(a\right)$ denotes the positive outranking and negative outranking flow for each alternative, respectively.

Step 7.5. Calculation of net outranking flow/ The PROMETHEE II complete ranking

(21) $$\varphi \left(a\right)=\varphi^{+}\left(a\right)-\varphi^{-}\left(a\right)$$

Where φ(a) denotes the net outranking flow for each alternative.

Step 8 – Definition of final ranking

In this step, we present the second novelty of this new method. In step 6, we present the matrix. The matrix $RWm_{ij}^{n}$ contains t sets of weights per criterion. The innovation point of this method is to generate t sets of rankings as different sets of weights are used, varying within the range of weights for each criterion, as dealt with in step 5. In this sense, φ(a) is transformed into an ordinal value. The φ(a) is sorted in descending order, being assigned 1st place to alternative (a) that has the highest φ(a), and so on until the last alternative m. The final ranking matrix FRm is of dimension t x m, where m is the number of columns composed of each alternative (a). Where t is the number of rows representing the ranking generated by the PROMETHEE method for each iteration. $a_{ij}$ is the ordinal value of the ranking that alternative j obtained in iteration i. As shown in Eq. (22):

(22) $$FRm_{ij}=a_{ij},\;\forall \;i=1,\;2,\;\ldots ,\;t\;and\;j=1,\;2,\;\ldots ,\;m$$

Then, the value of each rank-ordering $a_{ij}$will be replaced by a score, as follows: 1st = *m*, 2nd=(m-1), …, nth=(m-(m-1). Thus, the final ranking vector FRv of dimension j. The final position of each alternative will be obtained by summing the scores of the t iterations of each alternative. As shown in Eq. (23):

(23) $$FRv_{j}=\sum_{i=1}^{t}FRm_{ij},\;j=1,\;2,\;\ldots ,\;m$$

The final ranking will be obtained in descending order among the total scores of each alternative j of the vector $FRv_{j}$.

## Method validation

In this section we will use the data published in the research carried out by de Assis et al. [28], where they modelled the multi-criteria problem of choosing mobile rotorcraft for use in the police service in the city of Rio de Janeiro/Brazil. In this problem we will be working with 17 criteria and 15 alternatives. To solve this problem we will use the tool developed in Python by the authors and called EC-PROMETHEE, and available on the following website: https://pypi.org/project/ec-promethee/. The model's input data for validating the method is presented below:

*Data:*

*1) Criteria and Alternatives:*

Table 2 shows in column 1 the 15 alternatives used in the original model presented by de Assis et al. [28], and in column 2 the 17 criteria identified by the researchers are recorded.

**Table 2:** Alternatives and criteria established for analysis.

Alternatives		Criteria	
A1	Airbus H125 B3 (Squirrel)	C1	Price (US$)
A2	Airbus H125 B2 (Squirrel)	C2	Autonomy (minutes)
A3	Airbus H355 (Twin-Engine Squirrel)	C3	Speed VNE (Knots)
A4	Airbus EC 145 (BK-117 C2)	C4	Maximum number of people on board
A5	Airbus EC 135	C5	Versatility
A6	Airbus EC 120 (Hummingbird)	C6	Number of engines
A7	Bell UH-1H (Huey II)	C7	System redundancy
A8	Bell 206 (Long Ranger)	C8	Maximum take-off weight (Kg)
A9	Bell 412	C9	Payload (Kg)
A10	Bell 429	C10	Capacity for instrument flights
A11	Leonardo AW 119 Kx	C11	Autopilot
A12	Leonardo AW 139	C12	Embedded technology
A13	Robinson 44	C13	Length (meters)
A14	Robinson 66	C14	Engine power
A15	Sikorsky UH-60 (Black Hawk)	C15	Aftermarket
		C16	Availability
		C17	Protection

*Note: based on de Assis et al. [**28**].*

*2) Weights used in the original model:*

Table 3 describes the weights used in the original model by de Assis et al. [28].

**Table 3:** Matrix about the weights of the criteria.

Criteria	C1	C2	C3	C4	C5	C6	C7	C8	C9	C10	C11	C12	C13	C14	C15	C16	C17
Weight	0.047	0.065	0.053	0.062	0.070	0.058	0.060	0.066	0.066	0.046	0.042	0.058	0.048	0.066	0.059	0.068	0.066

*Note: based on de Assis et al. [**28**].*

*3) Matrix of evaluation:*

Table 4 shows the values corresponding to the alternatives in the model, evaluated criterion by criterion, which will be entered into the EC-PROMETHE tool. Table 5 shows the model's normalised data.

**Table 4:** Matrix of evaluation of the aircraft concerning the criteria.

Alternatives	Criteria																
	C1	C2	C3	C4	C5	C6	C7	C8	C9	C10	C11	C12	C13	C14	C15	C16	C17
A1	4826,857	200	155	6.0	4.7	1.0	3.0	2250	976	1.8	2.0	3.6	10.9	4.5	4.0	4.0	1.7
A2	1500,000	200	155	6.0	4.6	1.0	2.7	2250	1000	1.8	1.6	2.8	10.9	3.9	3.9	3.9	1.8
A3	1500,000	200	150	6.0	4.5	2.0	3.2	2600	930	2.2	3.0	3.2	11.0	3.5	3.5	3.7	1.7
A4	9000,000	210	150	11.0	3.8	2.0	4.0	3585	1905	3.5	3.5	3.8	13.0	3.3	3.3	4.0	1.3
A5	6000,000	216	136	8.0	3.8	2.0	4.0	2980	1418	3.5	3.5	3.8	12.3	3.3	3.3	5.0	1.3
A6	795,000	312	150	6.0	3.0	1.0	2.0	1715	755	2.0	1.0	3.0	9.6	2.5	3.0	4.0	1.5
A7	8420,000	120	130	13.0	4.2	1.0	2.4	4772	2300	2.4	2.2	2.8	13.3	3.9	2.3	2.1	4.3
A8	2000,000	222	130	7.0	3.0	1.0	1.0	1451	600	1.0	1.0	2.0	8.7	2.0	3.0	3.0	1.0
A9	6000,000	228	124	15.0	4.0	2.0	4.0	5400	2327	3.0	4.0	4.0	14.2	4.0	3.0	3.0	3.0
A10	7000,000	270	155	8.0	5.0	2.0	4.0	3402	1476	3.0	4.0	4.0	13.0	4.0	3.0	4.0	2.0
A11	3600,000	312	152	8.0	5.0	1.0	3.3	2850	908	3.0	3.0	4.3	13.0	4.0	4.0	4.0	4.0
A12	12,000,000	260	167	17.0	5.0	2.0	5.0	6800	2300	4.5	5.0	4.5	16.6	4.0	4.0	4.5	3.0
A13	450,000	200	130	4.0	2.5	1.0	1.0	1134	320	1.0	1.0	1.0	9.0	2.0	4.0	4.5	1.0
A14	1260,000	180	140	5.0	3.0	1.0	1.0	1225	420	1.0	1.0	3.0	9.0	3.0	3.0	4.0	1.0
A15	25,000,000	468	159	14.0	5.0	2.0	4.7	10,660	4100	4.6	3.8	4.4	20.0	5.0	4.3	4.8	5.0

*Note*: based on de Assis et al. [28].

**Table 5:** Aircraft evaluation matrix concerning criteria with normalized values.

Alternatives	Criteria																
	C1	C2	C3	C4	C5	C6	C7	C8	C9	C10	C11	C12	C13	C14	C15	C16	C17
A1	0.093	0.427	0.928	0.353	0.940	0.500	0.600	0.211	0.238	0.391	0.400	0.800	0.802	0.900	0.930	0.800	0.340
A2	0.300	0.427	0.928	0.353	0.920	0.500	0.540	0.211	0.244	0.391	0.320	0.622	0.802	0.780	0.907	0.780	0.360
A3	0.300	0.427	0.898	0.353	0.900	1.000	0.640	0.244	0.227	0.478	0.600	0.711	0.795	0.700	0.814	0.740	0.340
A4	0.050	0.449	0.898	0.647	0.760	1.000	0.800	0.336	0.465	0.761	0.700	0.844	0.672	0.660	0.767	0.800	0.260
A5	0.075	0.462	0.814	0.471	0.760	1.000	0.800	0.280	0.346	0.761	0.700	0.844	0.713	0.660	0.767	1.000	0.260
A6	0.566	0.667	0.898	0.353	0.600	0.500	0.400	0.161	0.184	0.435	0.200	0.667	0.910	0.500	0.698	0.800	0.300
A7	0.053	0.256	0.778	0.765	0.840	0.500	0.480	0.448	0.561	0.522	0.440	0.622	0.657	0.780	0.535	0.420	0.860
A8	0.225	0.474	0.778	0.412	0.600	0.500	0.200	0.136	0.146	0.217	0.200	0.444	1.000	0.400	0.698	0.600	0.200
A9	0.075	0.487	0.743	0.882	0.800	1.000	0.800	0.507	0.568	0.652	0.800	0.889	0.615	0.800	0.698	0.600	0.600
A10	0.064	0.577	0.928	0.471	1.000	1.000	0.800	0.319	0.360	0.652	0.800	0.889	0.672	0.800	0.698	0.800	0.400
A11	0.125	0.667	0.910	0.471	1.000	0.500	0.660	0.267	0.221	0.652	0.600	0.956	0.672	0.800	0.930	0.800	0.800
A12	0.038	0.556	1.000	1.000	1.000	1.000	1.000	0.638	0.561	0.978	1.000	1.000	0.527	0.800	0.930	0.900	0.600
A13	1.000	0.427	0.778	0.235	0.500	0.500	0.200	0.106	0.078	0.217	0.200	0.222	0.971	0.400	0.930	0.900	0.200
A14	0.357	0.385	0.838	0.294	0.600	0.500	0.200	0.115	0.102	0.217	0.200	0.667	0.971	0.600	0.698	0.800	0.200
A15	0.018	1.000	0.952	0.824	1.000	1.000	0.940	1.000	1.000	1.000	0.760	0.978	0.437	1.000	1.000	0.960	1.000

*Note*: based on de Assis et al. [28].

## Results

Stages 1 and 2 of the EC-PROMETHEE model were obtained using data from the research by de Assis et al. [26], which will be applied to exemplify the EC-PROMETHEE tool developed in Python by the authors. The proposed model allows the decision maker to use the weights generated objectively by the Entropy and CRITIC methods of the EC-PROMETHE model, or in addition to these, they can insert another set of weights from outside the model, which will make up the parameters for choosing the upper and lower limits of the weight ranges generated. This allows you to rely on the impartiality of objective methods for obtaining criteria weights, without losing the experience of experts, which permeates subjective methods for obtaining weights. In this example, in addition to the weights generated internally by the model, we chose to insert the weights used by de Assis et al. [26], which were obtained by a group of experts on the subject. In this way, we preserve the characteristics of the original model, which was obtained using the WASPAS muti-criteria method.

Therefore, we will illustrate below the step-by-step introduction of data into the environment of the EC-PROMETHEE python tool. The following code introduces the parameters to be used in the PROMETHEE method for ranking alternatives. The parameters are identified as follows:

*# Parameters for the PROMETHEE II Method*

*Q = [0.0, 0.0, 0.0, 0.0, 0.0, 0.0, 0.0, 0.0, 0.0, 0.0, 0.0, 0.0, 0.0, 0.0, 0.0, 0.0, 0.0]*

*S = [0.5, 0.5, 0.5, 0.5, 0.5, 0.5, 0.5, 0.5, 0.5, 0.5, 0.5, 0.5, 0.5, 0.5, 0.5, 0.5, 0.5]*

*P = [1.0, 1.0, 1.0, 1.0, 1.0, 1.0, 1.0, 1.0, 1.0, 1.0, 1.0, 1.0, 1.0, 1.0, 1.0, 1.0, 1.0]*

*F = ['t1′, 't1′, 't1′, 't1′, 't1′, 't1′, 't1′, 't1′, 't1′, 't1′, 't1′, 't1′, 't1′, 't1′, 't1′, 't1′, 't1′]*

*# 't1′ = Usual; 't2′ = U-Shape; 't3′ = V-Shape; 't4′ = Level; 't5′ = V-Shape with Indifference; 't6′ = Gaussian; 't7′ = C-Form*

*criterion_type = ['max', 'max', 'max', 'max', 'max', 'max', 'max', 'max', 'max', 'max', 'max', 'max', 'max', 'max', 'max', 'max', 'max']*

*iterations = 10,000*

The use of criteria weights external to the model is an option presented by EC-PROMETHEE to the decision maker in order to better discriminate the model. These weights can be obtained from experts, or even using other methods such as AHP, among others. The criteria weight data recorded in Table 3 was inserted into the Python tool code below:

*# OPTIONAL: User-defined Custom Weigths*

*custom_sets = [[0.047, 0.065, 0.053, 0.062, 0.070, 0.058, 0.060, 0.066, 0.066, 0.046, 0.042, 0.058, 0.048, 0.066, 0.059, 0.068, 0.066]]*

In step 3 of the EC-PROMETHE we entered the model evaluation matrix shown in Table 4. In this validation method, we chose to use the normalised evaluation matrix. As described in the following code:

*# Dataset*

*dataset = np.array([*

*[0.093, 0.427, 0.928, 0.353, 0.940, 0.500, 0.600, 0.211, 0.238, 0.391, 0.400, 0.800, 0.802, 0.900, 0.930, 0.800, 0.340],*

*[0.300, 0.427, 0.928, 0.353, 0.920, 0.500, 0.540, 0.211, 0.244, 0.391, 0.320, 0.622, 0.802, 0.780, 0.907, 0.780, 0.360],*

*[0.300, 0.427, 0.898, 0.353, 0.900, 1.000, 0.640, 0.244, 0.227, 0.478, 0.600, 0.711, 0.795, 0.700, 0.814, 0.740, 0.340],*

*[0.050, 0.449, 0.898, 0.647, 0.760, 1.000, 0.800, 0.336, 0.465, 0.761, 0.700, 0.844, 0.672, 0.660, 0.767, 0.800, 0.260],*

*[0.075, 0.462, 0.814, 0.471, 0.760, 1.000, 0.800, 0.280, 0.346, 0.761, 0.700, 0.844, 0.713, 0.660, 0.767, 1.000, 0.260],*

*[0.566, 0.667, 0.898, 0.353, 0.600, 0.500, 0.400, 0.161, 0.184, 0.435, 0.200, 0.667, 0.910, 0.500, 0.698, 0.800, 0.300],*

*[0.053, 0.256, 0.778, 0.765, 0.840, 0.500, 0.480, 0.448, 0.561, 0.522, 0.440, 0.622, 0.657, 0.780, 0.535, 0.420, 0.860],*

*[0.225, 0.474, 0.778, 0.412, 0.600, 0.500, 0.200, 0.136, 0.146, 0.217, 0.200, 0.444, 1.000, 0.400, 0.698, 0.600, 0.200],*

*[0.075, 0.487, 0.743, 0.882, 0.800, 1.000, 0.800, 0.507, 0.568, 0.652, 0.800, 0.889, 0.615, 0.800, 0.698, 0.600, 0.600],*

*[0.064, 0.577, 0.928, 0.471, 1.000, 1.000, 0.800, 0.319, 0.360, 0.652, 0.800, 0.889, 0.672, 0.800, 0.698, 0.800, 0.400],*

*[0.125, 0.667, 0.910, 0.471, 1.000, 0.500, 0.660, 0.267, 0.221, 0.652, 0.600, 0.956, 0.672, 0.800, 0.930, 0.800, 0.800],*

*[0.038, 0.556, 1.000, 1.000, 1.000, 1.000, 1.000, 0.638, 0.561, 0.978, 1.000, 1.000, 0.527, 0.800, 0.930, 0.900, 0.600],*

*[1.000, 0.427, 0.778, 0.235, 0.500, 0.500, 0.200, 0.106, 0.078, 0.217, 0.200, 0.222, 0.971, 0.400, 0.930, 0.900, 0.200],*

*[0.357, 0.385, 0.838, 0.294, 0.600, 0.500, 0.200, 0.115, 0.102, 0.217, 0.200, 0.667, 0.971, 0.600, 0.698, 0.800, 0.200],*

*[0.018, 1.000, 0.952, 0.824, 1.000, 1.000, 0.940, 1.000, 1.000, 1.000, 0.760, 0.978, 0.437, 1.000, 1.000, 0.960, 1.000] ])*

After entering the criteria, alternatives and evaluation matrix of the model into the EC-PROMETHEE tool, we began executing steps 4–5, where Eqs. (1)–(13) were run. Table 6 records the weights generated internally in the EC-PROMETHEE method, as well as illustrating the external weights inserted. The result of these steps is the definition of the upper and lower limits of the range of weights per criteria that will be used to obtain the total number of interactions to define the model's final ranking.

**Table 6:** Table with the weights generated and the definition of the upper and lower limits of the model's weight ranges.

Weight Name	g1	g2	g3	g4	g5	g6	g7	g8	g9	g10	g11	g12	g13	g14	g15	g16	g17
Entropy	0.295	0.027	0.002	0.052	0.013	0.033	0.058	0.116	0.116	0.058	0.072	0.026	0.014	0.017	0.007	0.01	0.084
Critic	0.125	0.04	0.049	0.05	0.056	0.081	0.041	0.044	0.043	0.04	0.045	0.049	0.122	0.042	0.056	0.059	0.057
Custom Weights 1	0.047	0.065	0.053	0.062	0.07	0.058	0.06	0.066	0.066	0.046	0.042	0.058	0.048	0.066	0.059	0.068	0.066
Lower	0.047	0.027	0.002	0.05	0.013	0.033	0.041	0.044	0.043	0.04	0.042	0.026	0.014	0.017	0.007	0.01	0.057
Upper	0.295	0.065	0.053	0.062	0.07	0.081	0.06	0.116	0.116	0.058	0.072	0.058	0.122	0.066	0.059	0.068	0.084

*Note*: Data output generated by the EC-PROMETHEE tool in python.

*# Show Weights Lower and Upper Bounds*

*df = ecp.weights_df*

*data_table.DataTable(df.round(3), num_rows_per_page = 15)*

Once the upper and lower limits of the weight ranges for each criterion have been obtained, the tool produces n sets of weights to be applied to the PROMETHEE ranking model. In this validation we used n = 10,000 iterations. Table 7 shows the output of the 10,000 sets of weights generated by running the code described below.

**Table 7:** Table with n iterations of the generated criteria bands.

index	g1	g2	g3	g4	g5	g6	g7	g8	g9	g10	g11	g12	g13	g14	g15	g16	g17
Iteration 1	0.195	0.053	0.007	0.057	0.04	0.047	0.059	0.049	0.07	0.053	0.057	0.054	0.116	0.032	0.055	0.025	0.074
Iteration 2	0.263	0.032	0.036	0.054	0.018	0.034	0.047	0.09	0.044	0.056	0.064	0.03	0.121	0.041	0.036	0.051	0.065
Iteration 3	0.047	0.029	0.009	0.05	0.033	0.038	0.043	0.051	0.103	0.053	0.044	0.05	0.044	0.038	0.034	0.036	0.064
Iteration 4	0.28	0.062	0.051	0.058	0.042	0.065	0.055	0.112	0.108	0.058	0.065	0.034	0.023	0.034	0.013	0.019	0.065
Iteration 5	0.054	0.032	0.037	0.055	0.025	0.039	0.056	0.101	0.106	0.042	0.062	0.04	0.117	0.052	0.046	0.06	0.064
Iteration 6	0.232	0.045	0.009	0.06	0.046	0.035	0.054	0.113	0.081	0.053	0.07	0.047	0.068	0.038	0.038	0.023	0.064
Iteration 7	0.148	0.055	0.006	0.061	0.065	0.064	0.049	0.052	0.114	0.048	0.052	0.043	0.054	0.026	0.037	0.051	0.076
Iteration 8	0.178	0.064	0.043	0.055	0.057	0.048	0.052	0.115	0.092	0.058	0.043	0.052	0.065	0.023	0.05	0.03	0.067
Iteration 9	0.093	0.065	0.045	0.055	0.017	0.05	0.044	0.097	0.103	0.04	0.064	0.038	0.038	0.063	0.046	0.042	0.07
Iteration 10	0.26	0.063	0.011	0.058	0.05	0.055	0.055	0.06	0.102	0.049	0.063	0.029	0.027	0.053	0.033	0.015	0.066
Iteration 11	0.129	0.047	0.019	0.061	0.023	0.044	0.054	0.07	0.093	0.044	0.061	0.035	0.025	0.057	0.053	0.066	0.083
Iteration 12	0.284	0.05	0.007	0.053	0.051	0.057	0.044	0.047	0.074	0.056	0.071	0.031	0.09	0.063	0.026	0.015	0.071
Iteration 13	0.094	0.036	0.026	0.052	0.015	0.055	0.053	0.069	0.116	0.056	0.054	0.039	0.036	0.055	0.048	0.013	0.082
Iteration 14	0.245	0.061	0.026	0.052	0.059	0.035	0.048	0.077	0.113	0.049	0.059	0.056	0.06	0.044	0.038	0.03	0.062
Iteration 15	0.264	0.034	0.019	0.061	0.018	0.037	0.053	0.092	0.083	0.041	0.062	0.027	0.116	0.042	0.019	0.023	0.059
…	…	…	…	…	…	…	…	…	…	…	…	…	…	…	…	…	…
Iteration 9991	0.215	0.029	0.048	0.059	0.04	0.036	0.043	0.107	0.104	0.052	0.062	0.045	0.056	0.044	0.031	0.049	0.072
Iteration 9992	0.196	0.062	0.012	0.054	0.047	0.066	0.051	0.096	0.082	0.041	0.05	0.053	0.027	0.052	0.036	0.06	0.059
Iteration 9993	0.144	0.043	0.04	0.054	0.067	0.073	0.049	0.115	0.104	0.056	0.05	0.039	0.039	0.056	0.026	0.061	0.066
Iteration 9994	0.066	0.047	0.017	0.056	0.014	0.053	0.06	0.095	0.075	0.055	0.056	0.029	0.082	0.025	0.058	0.024	0.066
Iteration 9995	0.057	0.054	0.051	0.06	0.019	0.036	0.049	0.074	0.064	0.051	0.063	0.046	0.111	0.055	0.036	0.02	0.078
Iteration 9996	0.274	0.03	0.051	0.061	0.019	0.053	0.049	0.1	0.074	0.057	0.057	0.043	0.027	0.031	0.026	0.013	0.082
Iteration 9997	0.111	0.042	0.034	0.055	0.046	0.061	0.046	0.098	0.076	0.043	0.054	0.044	0.108	0.057	0.016	0.054	0.067
Iteration 9998	0.242	0.029	0.048	0.05	0.033	0.081	0.047	0.111	0.055	0.053	0.048	0.026	0.075	0.037	0.021	0.015	0.083
Iteration 9999	0.195	0.033	0.039	0.051	0.061	0.041	0.044	0.078	0.063	0.044	0.044	0.054	0.076	0.059	0.03	0.012	0.074
Iteration 10,000	0.168	0.04	0.039	0.052	0.06	0.054	0.045	0.079	0.11	0.045	0.072	0.027	0.024	0.038	0.054	0.021	0.08

*Note*: Data output generated by the EC-PROMETHEE tool in python.



**Fig. 2:**
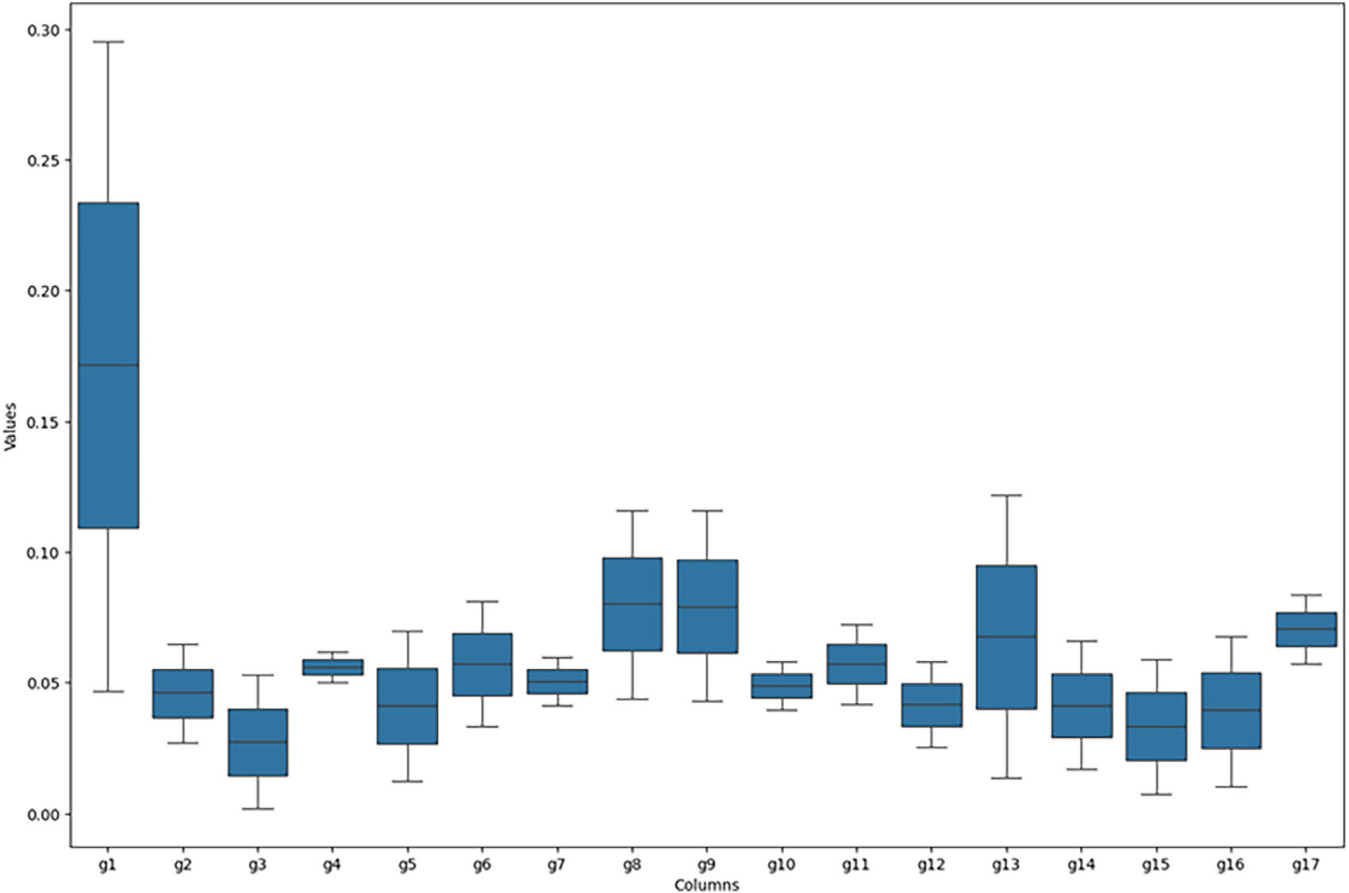
Graphical representation of the variation in the weight range for each criterion. *Note*: graphical output is generated by the EC-PROMETHEE tool in Python.



In addition to the table with the n iterations generated by the Python tool, the decision maker can graphically analyse the distribution of weights and the amplitude of the distribution for each criterion. Fig. 2 is one of the intermediate results of the process, to which the decision maker has access for analysis.

Next, we begin step 7, in which Eqs. (16)–(21) are run. Table 8 shows the *n* = 10,000 ranks generated in the model, based on the sets of weights in Table 7.

**Table 8:** Record of *n* = 10,000 ranks generated by EC-PROMETHEE.

index	a1	a2	a3	a4	a5	a6	a7	a8	a9	a10	a11	a12	a13	a14	a15
Iteration 1	9	8	6	10	7	11	12	15	4	5	3	2	13	14	1
Iteration 2	8	10	9	7	6	12	11	13	4	3	5	2	14	15	1
Iteration 3	8	11	9	7	6	12	10	14	4	3	5	2	15	13	1
Iteration 4	9	10	8	7	6	12	11	15	3	4	5	2	13	14	1
Iteration 5	9	10	8	7	6	12	11	14	3	5	4	2	13	15	1
Iteration 6	9	10	8	7	6	12	11	15	4	3	5	2	14	13	1
Iteration 7	9	10	8	7	6	12	11	13	4	3	5	2	14	15	1
Iteration 8	10	11	9	7	6	12	8	13	3	4	5	2	15	14	1
Iteration 9	9	10	8	7	6	12	11	13	4	3	5	2	14	15	1
Iteration 10	10	9	7	8	6	11	12	15	3	4	5	2	13	14	1
Iteration 11	10	8	7	9	6	11	13	15	3	4	5	2	12	14	1
Iteration 12	9	7	6	11	8	10	12	15	4	3	5	2	13	14	1
Iteration 13	9	11	8	7	6	12	10	13	3	4	5	2	14	15	1
Iteration 14	9	11	8	7	6	12	10	13	3	4	5	2	15	14	1
Iteration 15	9	10	7	8	6	11	12	15	3	4	5	2	13	14	1
…	…	…	…	…	…	…	…	…	…	…	…	…	…	…	…
Iteration 9991	9	11	8	7	6	10	12	15	4	3	5	2	13	14	1
Iteration 9992	9	10	8	6	7	12	11	13	3	4	5	2	14	15	1
Iteration 9993	9	10	8	7	6	12	11	15	3	4	5	2	14	13	1
Iteration 9994	9	10	7	8	6	11	12	15	5	3	4	2	13	14	1
Iteration 9995	10	8	6	11	7	9	13	15	4	5	3	2	12	14	1
Iteration 9996	11	9	7	8	6	10	12	15	3	5	4	2	13	14	1
Iteration 9997	11	8	7	9	6	10	12	15	3	4	5	2	13	14	1
Iteration 9998	11	9	8	7	6	12	10	15	3	4	5	2	13	14	1
Iteration 9999	8	10	9	7	5	12	11	14	3	4	6	2	15	13	1
Iteration 10,000	11	9	7	8	6	12	10	15	3	4	5	2	14	13	1

*Note*: Data output generated by the EC-PROMETHEE tool in python.

# Ranks Matrix

*dr = ecp.df_r*

*data_table.DataTable(dr.round(3), num_rows_per_page = 15)*

**Fig. 3:**
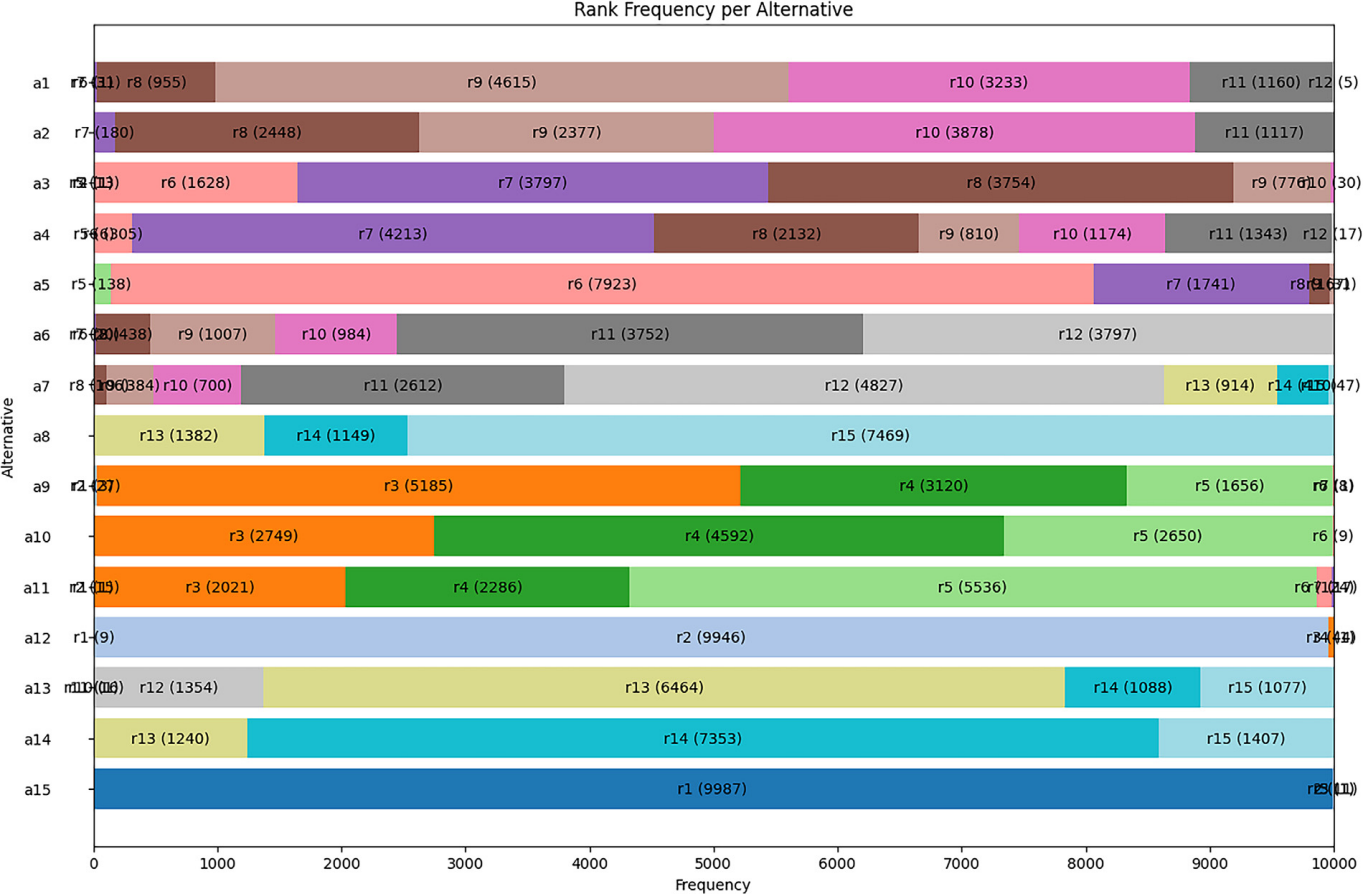
Graphical representation of the variation in the ranks occupied by the alternatives throughout the process. Graphical representation of the variation in the ranks occupied by the alternatives throughout the process. *Note*: graphical output is generated by the EC-PROMETHEE tool in Python.



The python tool presents a graphical solution in which researchers and/or decision-makers can objectively analyse the variations in the ranks occupied by the problem's solution alternatives as a function of the n iterations carried out. Fig. 3 illustrates the graphical representation of the n ranks recorded in Table 8. This figure clearly illustrates the influence of weight variation on the rank. This implementation works as a sensitivity analysis incorporated into EC-PROMETHEE.

**Fig. 4:**
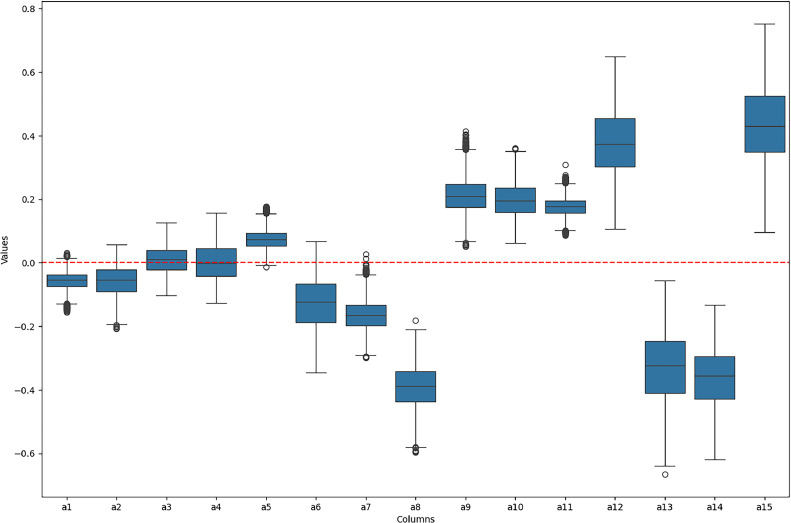
Boxplot with the distribution of the final flow of EC-PROMETHEE. *Note*: graphical output is generated by the EC-PROMETHEE tool in Python.

**Fig. 5:**
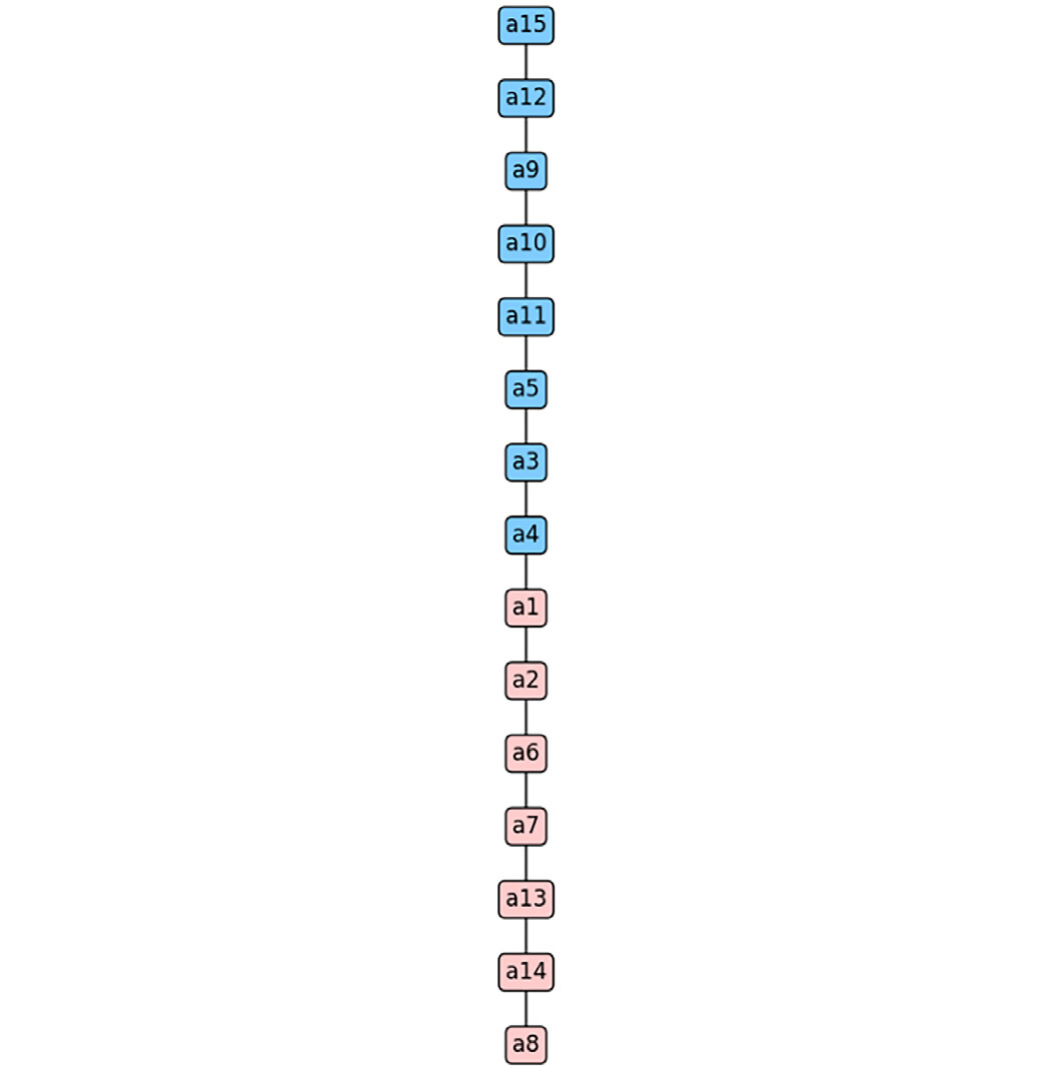
Graphical representation of the final rank of the EC-PROMETHEE method. *Note*: graphical output is generated by the EC-PROMETHEE tool in Python.



Finally, we begin step 8, where Eqs. (22) and (23), which define the final rank, are executed. The EC-PROMETHEE tool provides two outputs, the first being a list of the alternatives and their final positions in the rankings. The second is a graphical solution in which the alternatives are ordered according to their position in the final rank, and also presents a boxplot for analysing the layout of the final flow of the PROMETHEE method, as shown in Figs. 4 and 5.

## CRediT authorship contribution statement

**Marcio Pereira Basilio:** Conceptualization, Methodology, Writing – original draft, Writing – review & editing. **Valdecy Pereira:** Software, Data curation. **Fatih Yiğit:** Visualization, Investigation, Supervision.

## Declaration of competing interest

The authors declare that they have no known competing financial interests or personal relationships that could have appeared to influence the work reported in this paper.

## Data Availability

Data will be made available on request.

## Appendix Supplementary materials

Image, application 1

## References

[1] Basílio M.P., Pereira V., Costa H.G., Santos M., Ghosh A. (2022). A systematic review of the applications of multi-criteria decision aid methods (1977–2022). Electronics.

[2] Ayan B., Abacıoğlu S., Basilio M.P. (2023). A comprehensive review of the novel weighting methods for multi-criteria decision-making. Information.

[3] Saaty R.W. (1987). The analytic hierarchy process—what it is and how it is used. Math. Model..

[4] Singh M., Pant M. (2021). A review of selected weighing methods in MCDM with a case study. Int. J. Syst. Assur. Eng. Manag..

[5] Keršulienė V., Zavadskas E.K., Turskis Z. (2010). Selection of rational dispute resolution method by applying new step-wise weight assessment ratio analysis (Swara) by applying new step-wise weight assessment ratio. J. Bus. Econ. Manag..

[6] Mardani A., Nilashi M., Zakuan N., Loganathan N., Soheilirad S., Saman M.Z.M., Ibrahim O. (2017). A systematic review and meta-Analysis of SWARA and WASPAS methods: theory and applications with recent fuzzy developments. Appl. Soft Comput..

[7] Fernandes P.G., Quelhas O.L.G., Gomes C.F.S., Júnior E.L.P., Bella R.L.F., Junior C.D.S.R., Pereira R.C.A., Basilio M.P., dos Santos M. (2023). Product engineering assessment of subsea intervention equipment using SWARA-MOORA-3NAG method. Systems.

[8] Mukhametzyanov I. (2021). Specific character of objective methods for determining weights of criteria in MCDM problems: entropy, CRITIC and SD. Decis. Making Appl. Manag. Eng..

[9] Krishnan A.R., Kasim M.M., Hamid R., Ghazali M.F. (2021). A modified CRITIC method to estimate the objective weights of decision criteria. Symmetry.

[10] Rezaei J. (2015). Best-worst multi-criteria decision-making method. Omega.

[11] Zavadskas E.K., Podvezko V. (2016). Integrated determination of objective criteria weights in MCDM. Int. J. Inf. Technol. Decis. Mak..

[12] Pamučar D., Stević Ž., Sremac S. (2018). A new model for determining weight coefficients of criteria in Mcdm models: full consistency method (Fucom). Symmetry.

[13] Khan F., Ali Y., Pamucar D. (2022). A new fuzzy FUCOM-QFD approach for evaluating strategies to enhance the resilience of the healthcare sector to combat the COVID-19 pandemic. Kybernetes.

[14] Ižović M., Pamucar D. (2019). New model for determining criteria weights: level based weight assessment (LBWA). Model. Decis. Mak. Appl. Manag. Eng..

[15] Tenório F.M., Moreira M.L., Costa I.P.D.A., Gomes C.F.S., dos Santos M., Silva F.C.A., da Silva R.F., Basilio M.P. (2022). SADEMON: the computational web platform to the SAPEVO-M method. Procedia Comput. Sci..

[16] Keshavarz-Ghorabaee M., Amiri M., Zavadskas E.K., Turskis Z., Antucheviciene J. (2021). Determination of objective weights using a new method based on the removal effects of criteria (MEREC). Symmetry.

[17] Banik B., Alam S., Chakraborty A. (2023). Comparative study between GRA and MEREC technique on an agricultural-based MCGDM problem in pentagonal neutrosophic environment. Int. J. Environ. Sci. Technol..

[18] Biswas S., Joshi N. (2023). A performance based ranking of initial public offerings (IPOs) in India. J. Decis. Anal. Int. Comp..

[19] Ali J., Naser Hussain K., Alnoor A., Muhsen Y.R., Atiyah A.G. (2024). Benchmarking methodology of banks based on financial sustainability using CRITIC and RAFSI techniques. Decis. Mak. Appl. Manag. Eng..

[20] Basilio M.P., Pereira V., Yigit F. (2023). New hybrid EC-promethee method with multiple iterations of random weight ranges: applied to the choice of policing strategies. Mathematics.

[21] Mareschal B., Brans J.P. (1988). Geometrical representations for MCDA. Eur. J. Oper. Res..

[22] Brans J.P., Mareschal B. (1994). The PROMCALC & GAIA decision support system for multicriteria decision aid. Decis. Support Syst..

[23] Zolfani S.H., Taheri H.M., Gharehgozlou M., Farahani A. (2022). An asymmetric PROMETHEE II for cryptocurrency portfolio allocation based on return prediction. Appl. Soft Comput..

[24] Wei Q., Zhou C., Liu Q., Zhou W., Huang J. (2023). A barrier evaluation framework for forest carbon sink project implementation in China using an integrated BWM-IT2F-PROMETHEE II method. Expert Syst. Appl..

[25] Ajin M., Moses J., Dharshini M.P. (2023). Tribological and machining characteristics of AA7075 hybrid composites and optimizing utilizing modified PROMETHEE approach. Mater. Res. Express.

[26] Basilio M.P., Pereira V., de Oliveira M.W.C., Neto A.F.D.C. (2021). Ranking policing strategies as a function of criminal complaints: application of the PROMETHEE II method in the Brazilian context. J. Model. Manag..

[27] Basilio M.P., Pereira V. (2020). Operational research applied in the field of public security: the ordering of policing strategies such as the ELECTRE IV. J. Model. Manag..

[28] de Assis G.S., dos Santos M., Basilio M.P. (2023). Use of the WASPAS method to select suitable helicopters for aerial activity carried out by the military police of the state of Rio de Janeiro. Axioms.

