# Supplementary material for: New hybrid EC-PROMETHEE method with multiple iterations of random weight ranges: Step-by-step application in Python
Source: MethodsX. 2024 Aug 5;13:102890. doi: 10.1016/j.mex.2024.102890 (PMC11372792; doi:10.1016/j.mex.2024.102890)
Supplement: Supplementary file 1 [file mmc1.pdf]

REPÚBLICA FEDERATIVA DO BRASIL
MINISTÉRIO DO DESENVOLVIMENTO, INDÚSTRIA, COMÉRCIO E SERVIÇOS
INSTITUTO NACIONAL DA PROPRIEDADE INDUSTRIAL
DIRETORIA DE PATENTES, PROGRAMAS DE COMPUTADOR E TOPOGRAFIAS DE CIRCUITOS

Certificado de Registro de Programa de Computador

Processo Nº: **BR512023003272-7**

O Instituto Nacional da Propriedade Industrial expede o presente certificado de registro de programa de computador, válido por 50 anos a partir de 1° de janeiro subsequente à data de 30/10/2023, em conformidade com o §2°, art. 2° da Lei 9.609, de 19 de Fevereiro de 1998.

Título: The EC-PROMETHEE Method - A Committee Approach for Outranking Problems Using Randoms Weights

Data de publicação: 30/10/2023

Data de criação: 30/10/2023

Titular(es): VALDECY PEREIRA; MÁRCIO PEREIRA BASÍLIO; FATIH YIGIT

Autor(es): VALDECY PEREIRA; MÁRCIO PEREIRA BASÍLIO; FATIH YIGIT

Linguagem: PYTHON

Campo de aplicação: AD-01; AD-04; AD-05

Tipo de programa: AP-01

Algoritmo hash: SHA-512

Resumo digital hash:
54AD16AA7F51688C0556D523BC6DDD7C6F73EB66FA80EDFAF67DBB7DE939D4244234C6457DFE56988DA9235
9FACACD9B7402AB3AD8EDE6F41E3E0A2828646ED1

Expedido em: 07/11/2023

Aprovado por:
Carlos Alexandre Fernandes Silva
Chefe da DIPTO
